# Reflux symptom burden and objective findings at long-term follow-up after peroral endoscopic myotomy

**DOI:** 10.1007/s00464-026-13205-6

**Published:** 2026-08-03

**Authors:** Natalie N. Chakraborty, Emily F. Simon, Julia Specht, Joshua Lyons, Ayesha Siddiq, Maham Babur, Kayvan Barekatain, Trisha Lal, Christina S. Boutros, Patrick Wieland, Saher-Zahra Khan, Hamza Chatha, Jamie Benson, Nicolette Winder, Jeffrey M. Marks

**Affiliations:** 1 Department of Surgery, University Hospitals Cleveland Medical Center, 11100 Euclid Ave, Cleveland, OH 44106 USA; 2 Case Western Reserve University School of Medicine, Cleveland, OH USA; 3 Department of Medicine, University Hospitals Cleveland Medical Center, Cleveland, OH USA; 4 Department of Surgery, New York University, New York, NY USA

**Keywords:** Peroral endoscopic myotomy, Esophageal achalasia, Gastroesophageal reflux, Proton pump inhibitors

## Abstract

**Background:**

Peroral endoscopic myotomy (POEM) effectively treats achalasia but confers a significant risk of postoperative gastroesophageal reflux. Studies demonstrate poor short-term correlation between reflux symptoms and objective findings, but long-term outcomes remain unclear. We evaluated long-term GERD Health-Related Quality of Life (GERD-HRQL) scores, Bravo pH results, and proton pump inhibitor (PPI) use after POEM.

**Methods:**

We conducted a prospective survey study of adults who underwent POEM at our institution from 2011 to 2024 with > 1 year of follow-up. Standardized surveys included the validated GERD-HRQL questionnaire and questions regarding PPI use and post-POEM interventions. Chart review verified medication use, Bravo results, and endoscopic findings. The primary endpoint was reflux symptom control, defined as GERD-HRQL score < 10. Secondary endpoints included chronic PPI use (> 6 months), Bravo results, and the reflux–dysphagia relationship. Multivariable logistic regression identified predictors of reflux symptom control.

**Results:**

Seventy-nine patients completed the questionnaire at a median follow-up of 68 months. The median GERD-HRQL score was 10 (IQR 4–17), with 42 patients (53.2%) reporting clinically significant reflux (GERD-HRQL ≥ 10). Most (58; 73.4%) had chronic PPI use, 55.2% of whom reported poor symptom control. Among patients with GERD-HRQL ≥ 10, 52.4% had Eckardt scores > 3, suggesting concurrent dysphagia. Thirty-one patients underwent post-POEM Bravo; results were negative in 13 (41.9%), positive in 13 (41.9%), and inconclusive in 5 (16.1%). Six of 10 Bravos performed for reflux symptoms were negative. Endoscopy (*n* = 56) demonstrated esophagitis in 16 patients (28.6%). Older age (OR 1.04; 95% CI 1.01–1.08) and preoperative weight loss ≥ 5 kg (OR 6.72; 95% CI 1.57–28.74) predicted reflux control on multivariable analysis.

**Conclusions:**

At long-term follow-up, over half of patients reported clinically significant reflux, and most required chronic PPI therapy. Reflux symptoms correlated inconsistently with objective findings, mirroring short-term data and highlighting the need for ongoing surveillance and targeted reflux mitigation after POEM.

**Supplementary Information:**

The online version contains supplementary material available at https://doi.org/10.1007/s00464-026-13205-6.

Achalasia is an esophageal dysmotility disorder characterized by impaired relaxation of the lower esophageal sphincter (LES) and the absence of esophageal peristalsis [1]. Patients typically present with progressive dysphagia, chest pain, regurgitation of solid food, and weight loss [2]. Treatment is not curative and is directed at symptom improvement though reduction of LES pressure, which can be achieved through pneumatic dilation, peroral endoscopic myotomy (POEM), or surgical myotomy [1]. POEM has demonstrated superior short-term symptom resolution compared to pneumatic dilation, with up to a 92% success rate at 2 years [3, 4], and is equivalent to laparoscopic Heller myotomy with fundoplication regarding symptom control [5]. However, due to endoscopic LES disruption without a concurrent anti-reflux mechanism, gastroesophageal reflux disease (GERD) after POEM remains a significant concern, with abnormal acid exposure and esophagitis occurring in 41–65% of patients post-treatment [6].

Despite the established efficacy of POEM for dysphagia relief, long-term data on GERD-related outcomes remain limited [6, 7]. Poor correlation exists between subjective reflux symptoms and objective findings on pH monitoring or endoscopy, with up to one-quarter of patients with pathologic acid exposure remaining asymptomatic [8]. The efficacy of proton pump inhibitors (PPIs) in this population also remains unclear; while some studies report up to an 81% improvement in erosive esophagitis, correlation with patient-reported symptom improvement varies [9, 10]. Additionally, long-term data beyond 5 years are sparse, and existing studies demonstrate conflicting results regarding whether reflux symptoms improve or persist over time [7, 11]**.**

We therefore conducted a prospective, survey-based study of adults who underwent POEM for achalasia with long-term follow-up. We aimed to characterize the burden of reflux symptoms using validated questionnaires, assess the correlation between subjective symptoms and objective testing, and identify potential predictors of reflux symptom control to inform surveillance strategies and patient counseling.

## Materials and methods

### Patient cohort and institutional review board approval

Institutional Review Board approval (IRB Protocol: STUDY20241468) was obtained at University Hospitals Cleveland Medical Center. We retrospectively identified adult patients aged 18–100 years who underwent POEM at our institution between January 2011 and March 2024 and conducted a prospective, survey-based cohort study of post-POEM outcomes and interventions. Patients were excluded if they had less than one year of follow-up, if their index procedure in our database represented a repeat myotomy, if they were deceased at the time of contact, or if they were unable to consent.

Eligible patients were contacted (1) via an email containing a link to an online survey created using REDCap, a secure, web-based database platform, or (2) via a mailed letter, if no email address was available. Prior to survey completion, all participants reviewed an information sheet and provided verbal attestation permitting additional data collection from the electronic medical record (EMR). Nonresponders were contacted by telephone after 14 days (email) or 21 days (mailed letter), with two call attempts made; verbal consent was obtained prior to questionnaire administration. Standardized questionnaires, including the Eckardt score and Gastroesophageal Reflux Disease Health-Related Quality of Life (GERD-HRQL) surveys, along with questions regarding interval Bravo pH testing, proton pump inhibitor (PPI) use, and other procedures performed for achalasia, were administered. The standardized survey is included as Supplementary File 1. The Eckardt score is a validated 4-item instrument assessing dysphagia, regurgitation, chest pain, and weight loss (score range 0–12), with scores > 3 indicating treatment failure [9, 12]. The GERD-HRQL is a validated 10-item questionnaire evaluating heartburn severity and its impact on daily activities (score range 0–50) [13]. Survey responses were recorded directly into REDCap, with supplemental data, including verified medication use, Bravo pH results, and endoscopic findings, abstracted from the EMR. At our institution, routine Bravo pH studies are obtained 6 months post-POEM when feasible, or later for persistent reflux symptoms.

### Variables and outcomes of interest

Sociodemographic variables included age at POEM and sex. Clinical variables extracted from the EMR included the Charlson Comorbidity Index (0, 1–2, or 3 +), preoperative body mass index (BMI; underweight/normal weight: < 18.5–24.9 kg/m^2^, overweight: 25.0–29.9 kg/m^2^, or obese: ≥ 30.0 kg/m^2^), preoperative weight loss attributable to dysphagia (< 5 kg, ≥ 5 kg, or unknown), preoperative length of symptoms (< 1 year, 1–3 years, or ≥ 4 years), preoperative PPI use, preoperative GERD diagnosis, GERD diagnosis at 6 months post-POEM, preoperative Eckardt score, and Bravo and endoscopic results. Long-term variables collected via the survey included GERD-HRQL score, Eckardt score, interval to symptom return after POEM, any subsequent Bravo pH studies or achalasia-related procedures, and chronic PPI use (defined as > 6 months duration) since POEM. Covariates included in multivariable models were chosen a priori.

The primary endpoint was gastroesophageal reflux symptom control, defined as GERD-HRQL score < 10, with scores ≥ 10 considered indicative of clinically significant reflux symptoms. This threshold was selected based on surgical anti-reflux literature demonstrating that successfully treated patients typically achieve GERD-HRQL scores below 10, with median postoperative scores ranging from 0 to 5 [14–16]. While no standardized threshold exists for the GERD-HRQL questionnaire [13], a score of 10 represents a clinically meaningful departure from expected postintervention symptom improvement and corresponded to the median score in our cohort, providing a natural inflection point for stratification.

Secondary endpoints included Bravo pH monitoring and endoscopic findings after POEM, the frequency of chronic PPI use, additional interventions required for achalasia, and characterization of the relationship between reflux and dysphagia.

### Statistical analysis

Cohort characteristics were summarized and reported as frequencies with percentages for categorical variables or as medians with interquartile ranges (IQR) for continuous variables. Comparisons across groups, stratified by GERD-HRQL scores, were performed using Chi-square tests for categorical variables and Wilcoxon rank-sum tests for continuous variables. Multivariable logistic regression was used to determine predictors of reflux symptom control, with effect estimates presented as odds ratios (ORs) with 95% confidence intervals (95% CIs). A box-and-whisker plot was generated to visualize the distribution of long-term GERD-HRQL scores, displaying the median, interquartile range, and outliers.

All statistical analysis was performed using Stata 17 (StataCorp. 2021. *Stata Statistical Software: Release 17.* College Station, TX: StataCorp LLC)*.* An alpha level of < 0.05 was used to denote statistical significance. *P* values were derived from two-tailed tests.

## Results

### Patient cohort and follow-up

**Fig. 1:**
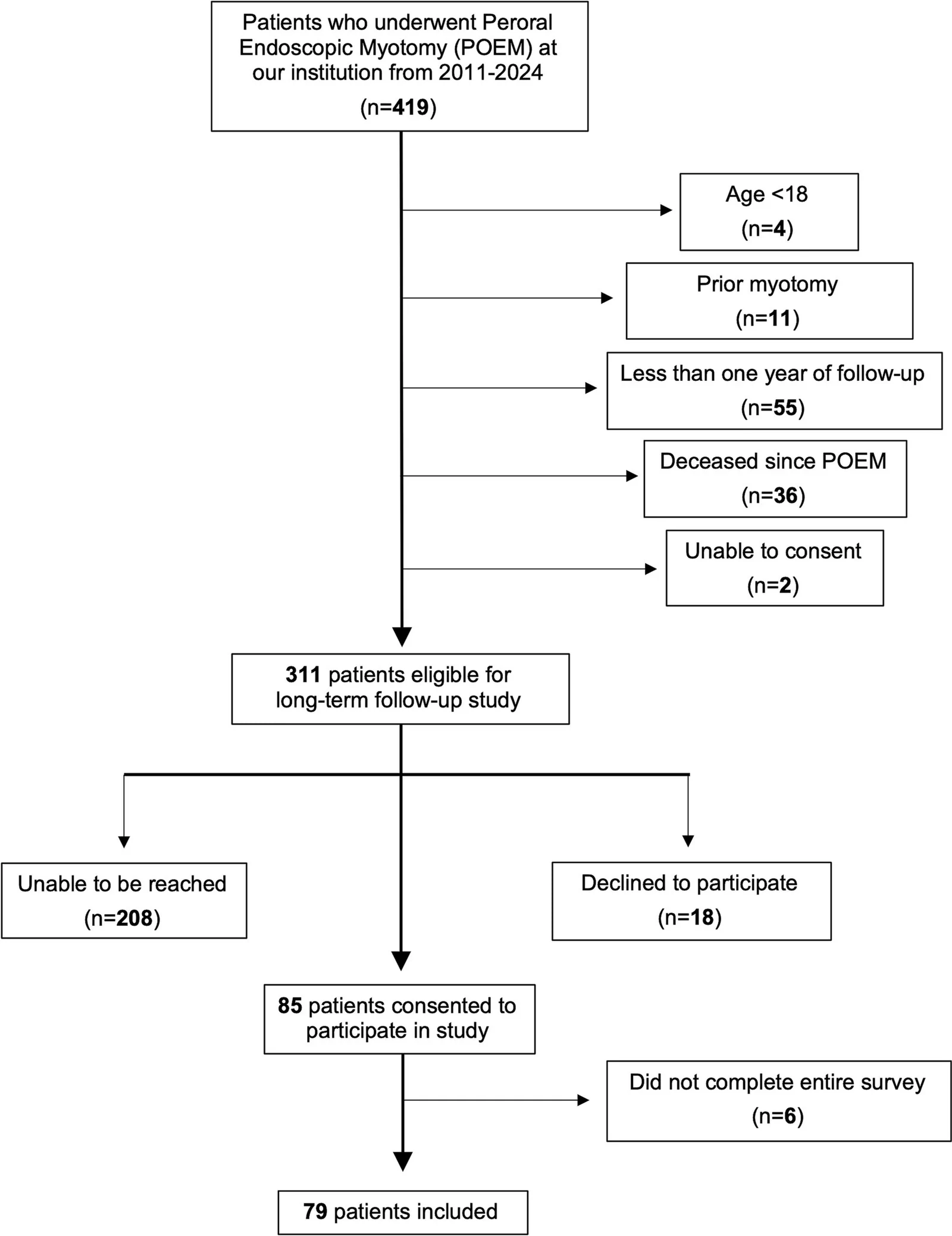
419 patients who underwent peroral endoscopic myotomy (POEM) at our institution from 2011 to 2024 were screened for eligibility. Exclusions included age < 18 years (*n* = 4), prior myotomy (*n* = 11), < 1 year follow-up (*n* = 55), death (*n* = 36), or inability to consent (*n* = 2), leaving 311 patients eligible. Of these, 208 could not be reached, and 18 declined participation. 85 consented, with 79 (25.4%) completing all survey items

Between 2011 and 2024, a total of 419 patients underwent POEM at our institution. Of these, 108 were ineligible for study participation: 4 due to age < 18 years, 11 due to undergoing repeat myotomy, 55 due to follow-up duration < 1 year, 36 who were deceased at the time of contact, and 2 due to inability to consent. This resulted in 311 patients meeting inclusion criteria for study participation, of whom 79 (25.4%) completed the survey (Fig. 1). Compared to patients who did not complete the survey, patients who completed the survey were more likely to be overweight or obese (*p* = 0.022), with no other significant baseline differences between nonrespondents and respondents. The median follow-up was 68 months (IQR 34–103 months).

Baseline cohort characteristics are presented in Table 1, stratified by long-term GERD-HRQL scores. Compared to patients with scores ≥ 10, patients with clinical reflux symptom control after POEM were older (median age 62.9 vs 52.2; *p* = 0.023) and more likely to have lost ≥ 5 kg preoperatively (35% vs 10%; *p* = 0.016). Other characteristics were balanced between groups (Table 1).

**Table 1:** Cohort characteristics according to long-term GERD-HRQL score after peroral endoscopic myotomy

Baseline variable	All respondents (*n* = 79)	GERD-HRQL score < 10 (*n* = 37)	GERD-HRQL score ≥ 10 (*n* = 42)	*P* value
Age: years, median (IQR)	59.2 (42.9 – 69.2)	62.9 (47.3–73.1)	52.2 (41.4–67.4)	0.023
Sex, No. (%)				0.23
Female	42 (53)	17 (46)	25 (60)	
Male	37 (47)	20 (54)	17 (40)	
Charlson Comorbidity Index, No. (%)				0.17
0	25 (32)	9 (24)	16 (38)	
1–2	22 (28)	9 (24)	13 (31)	
3+	32 (40)	19 (51)	13 (31)	
Preoperative BMI, No. (%)				0.37
Underweight/normal weight (< 18.5–24.9 kg/m^2^)	14 (18)	8 *(22)*	6 (14)	
Overweight (25.0–29.9 kg/m^2^)	20 (25)	11 (30)	9 (21)	
Obese (≥ 30 kg/m^2^)	45 (57)	18 (49)	27 (64)	
Preoperative weight loss, No. (%)				0.016
< 5 kg	45 (57)	16 (43)	29 *(69)*	
≥ 5 kg	17 (22)	13 (35)	4 (10)	
Unknown	17 (22)	8 (22)	9 (21)	
Preoperative symptom length, No. (%)				0.63
< 1 year	25 (32)	13 (35)	12 (29)	
1–3 years	23 (29)	12 (32)	11 (26)	
≥ 4 years	25 (32)	9 (24)	16 (38)	
Unknown	6 (7)	3 (8)	3 (7)	
Preoperative PPI use, No. (%)				0.85
No	28 (35)	13 (35)	15 (36)	
Yes	42 (53)	19 (51)	23 (55)	
Unknown	9 (11)	5 (14)	4 (10)	
Preoperative GERD diagnosis, No. (%)				0.91
No	55 (70)	26 (70)	29 (69)	
Yes	24 (30)	11 (30)	13 (31)	
GERD diagnosis at 6 months post-POEM, No. (%)				0.58
No	71 (90)	34 (92)	37 (88)	
Yes	8 (10)	3 (8)	5 (12)	
Preoperative Eckardt score, No. *(%)*				0.82
≤ 3	29 (37)	13 (35)	16 (38)	
> 3	45 (57)	21 (57)	24 (57)	
Unknown	5 (6)	3 (8)	2 (5)	
Myotomy location, No. (%)				0.50
Anterior	69 (87)	34 (92)	35 (83)	
Posterior	6 (8)	2 (5)	4 (10)	
Unknown	4 (5)	1 (3)	3 (7)	

*GERD-HRQL* Gastroesophageal Reflux Disease Health-Related Quality of Life, *IQR* interquartile range, *No* number, *BMI* body mass index, *PPI* proton pump inhibitor

### Reflux symptom control and GERD-HRQL score distribution

**Fig. 2:**
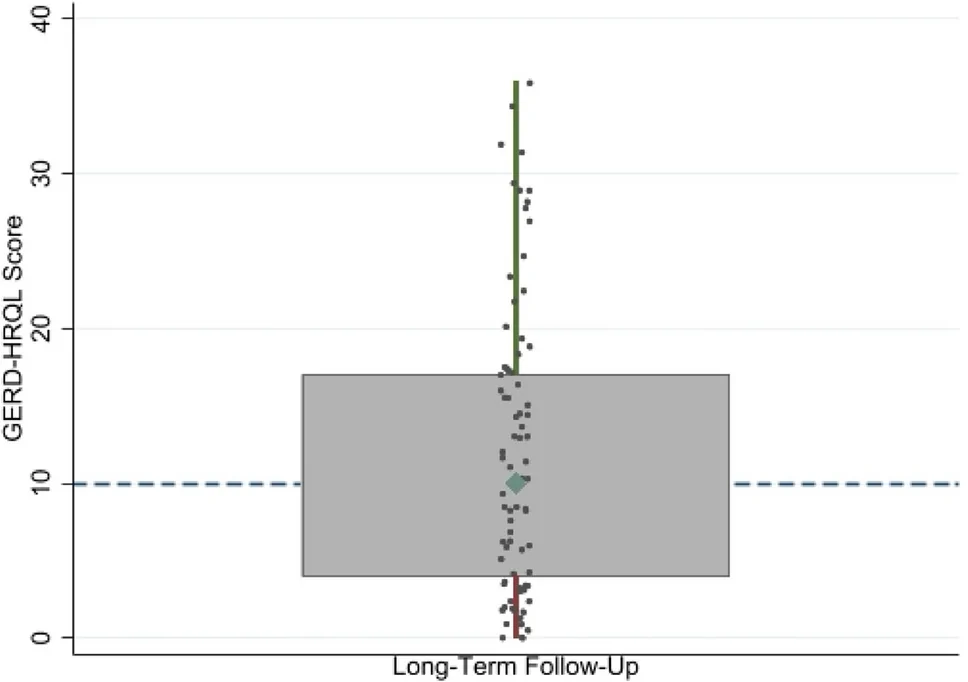
Box-and-whisker plot showing distribution of Gastroesophageal Reflux Disease Health-Related Quality of Life (GERD-HRQL) scores at long-term follow-up after POEM. Individual scores are displayed as overlaid points. The box represents the interquartile range (IQR), with the median indicated by the horizontal dashed line and the mean by the diamond marker. Whiskers represent the range of observed values

Forty-two (53.2%) patients endorsed clinically significant reflux symptoms (GERD-HRQL score ≥ 10) at long-term follow-up. GERD-HRQL scores demonstrated a broad distribution (Fig. 2). The median score was 10 (IQR 4–17). Individual scores ranged from 0 to 36 out of 50, indicating variable symptom burden at follow-up.

On multivariable analysis, older age (OR 1.04; 95% CI 1.01–1.08) and preoperative weight loss ≥ 5 kg attributable to dysphagia (OR 6.72; 95% CI 1.57–28.74) emerged as predictors of clinical reflux symptom control after POEM. No additional factors, including preoperative PPI use and preoperative GERD diagnosis, predicted long-term GERD-HRQL score (Table 2).

**Table 2:** Factors associated with clinical reflux symptom control after peroral endoscopic myotomy

Variable	Odds ratio	95% confidence interval	*P* value
Age	1.05	1.01–1.09	0.011
Sex			
Female	Ref	Ref	Ref
Male	2.04	0.66–6.28	0.215
Preoperative BMI			
Underweight/normal weight (< 18.5–24.9 kg/m^2^)	Ref	Ref	Ref
Overweight (25.0–29.9 kg/m^2^)	0.99	0.18–5.30	0.987
Obese (≥ 30 kg/m^2^)	0.52	0.11–2.43	0.405
Preoperative weight loss			
< 5 kg	Ref	Ref	Ref
> 5 kg	7.23	1.59–32.88	0.010
Unknown	1.32	0.56–4.92	0.676
Preoperative symptom length			
< 1 year	Ref	Ref	Ref
1–3 years	1.59	0.40–6.25	0.509
≥ 4 years	0.73	0.18–2.96	0.663
Unknown	1.64	0.19–14.01	0.652
Preoperative PPI use			
No	Ref	Ref	Ref
Yes	1.17	0.33–4.16	0.804
Unknown	3.00	0.35–25.66	0.317
Preoperative GERD diagnosis			
No	Ref	Ref	Ref
Yes	0.74	0.20–2.75	0.655
Myotomy location			
Anterior	Ref	Ref	Ref
Posterior	1.89	0.25–14.34	0.539
Unknown	0.15	0.01–2.52	0.189

*BMI* body mass index, *PPI* proton pump inhibitor, *GERD* gastroesophageal reflux disease

### Post-POEM interventions and objective findings

**Fig. 3:**
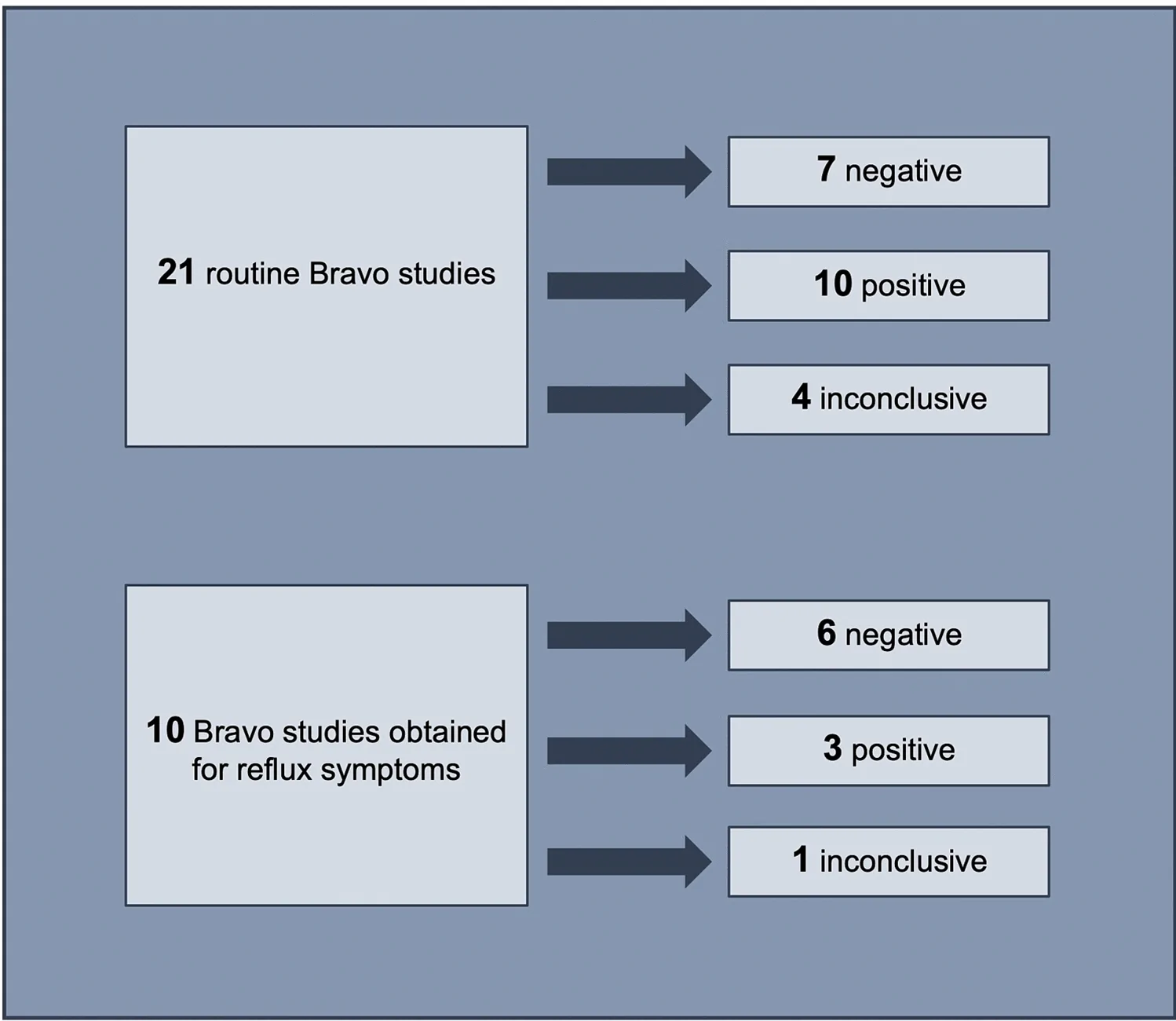
Summary of Bravo pH monitoring results after POEM. 21 studies were obtained as routine 6-month surveillance, yielding 7 negative, 10 positive, and 4 inconclusive results. An additional 10 studies were performed for evaluation of reflux symptoms, with 6 negative, 3 positive, and 1 inconclusive result. This figure compares objective results in routine surveillance versus symptom-driven evaluation after POEM

Thirty-one patients underwent Bravo pH monitoring after POEM. Among 21 routine studies performed at 6 months, 10 (47.6%) were positive, 7 (33.3%) were negative, and 4 (19.0%) were inconclusive. Of 10 Bravo studies obtained for evaluation of persistent reflux symptoms, only 3 (30%) were positive, while 6 (60%) were negative (Fig. 3). Fifty-six patients underwent endoscopy during follow-up, and esophagitis was identified in 16 (28.6%). Despite chronic PPI use in most patients (58; 73.4%), over half (55.2%) reported poor reflux symptom control.

Twenty-three patients underwent additional procedures for achalasia during the follow-up period; while most (16; 69.6%) required a single intervention, 7 required multiple procedures (range 2–4). Procedures included endoscopic lower esophageal sphincter (LES) dilation (*n* = 12), Botox injection (*n* = 10), repeat POEM (*n* = 8), Heller myotomy (*n* = 4), and esophagectomy (*n* = 1).

### Relationship between reflux and dysphagia symptoms

Among patients with significant reflux symptoms (GERD-HRQL ≥ 10), over half (52.4%) also reported persistent dysphagia (Eckardt > 3). Conversely, only 7 of 37 (18.9%) patients with reflux symptom control had Eckardt scores > 3 (*p* = 0.002).

## Discussion

This prospective cohort study evaluated long-term gastroesophageal reflux symptoms and objective findings at a median follow-up of 68 months after POEM, among the longest reported in the literature [17–19]. Over half of patients (53.2%) reported clinically significant reflux symptoms (GERD-HRQL ≥ 10), and nearly three-quarters required chronic PPI therapy, though symptom control remained suboptimal in the majority. These findings extend prior short-term observations underscoring the persistent burden of reflux symptoms following POEM [18, 19]. Individual GERD-HRQL scores varied widely (range 0–36), with most clustering in the lower to middle range but a subset demonstrating substantially higher scores, reflecting variability in long-term symptom burden.

The prevalence of reflux symptoms in our cohort exceeds pooled rates of 19–26% reported in prior meta-analyses [17, 20]. This difference may partly reflect our longer follow-up period. While Vespa et al. reported a median follow-up of 4 years, many studies in these analyses had follow-up under 2 years, some as brief as 3 months [17, 20]. Additionally, methodological heterogeneity may contribute, as many studies relied on subjective reflux endorsement rather than on validated questionnaires [18–20]. Our use of the GERD-HRQL questionnaire, which captures both symptom severity and impact on daily activities, may provide a more comprehensive assessment. Our observed esophagitis rate of 28.6% aligns closely with the pooled rate of 29.4% reported in a large meta-analysis [17]. A 2024 American Gastroenterological Association (AGA) clinical practice update confirms that abnormal acid exposure and esophagitis are common after POEM, ranging from 41 to 56% and 41 to 65%, respectively [4–6]. Notably, a cohort study of almost 3000 patients found that although reflux esophagitis occurred in 64.9% of patients, severe esophagitis (Los Angeles grade C/D) was uncommon at 7.5% [11]. Regardless, objective evaluation of pathologic acid exposure, typically around 6–12 months postoperatively, is suggested in all patients after POEM [6].

A key finding of our study is the discordance between reflux symptoms and objective testing. Among patients undergoing Bravo for persistent reflux symptoms, 60% had negative studies, whereas nearly half (47.6%) of asymptomatic patients undergoing routine monitoring demonstrated pathologic acid exposure. This mirrors existing literature demonstrating poor correlation between symptoms and pathologic acid exposure after POEM; one study found that among patients with objective GERD (DeMeester score > 14.7 or Los Angeles grade C/D esophagitis), 24% were asymptomatic [8, 18, 21]. Symptoms may also arise from etiologies beyond acid reflux, including non-reflux esophageal acidification from food stasis and visceral hypersensitivity [6, 22]. Overall, these findings underscore the limitations of symptom-based assessment alone and support guideline recommendations for surveillance with routine pH monitoring and endoscopy after POEM. It is our practice to obtain routine Bravo pH monitoring approximately 6 months after POEM when feasible. However, objective surveillance is sometimes limited by loss to follow-up, as evidenced by only 39.2% of survey respondents undergoing post-POEM Bravo, likely reflecting symptom improvement and our broad geographic referral base. In accordance with AGA recommendations, we also advise surveillance endoscopy every 3–4 years after POEM, with earlier evaluation reserved for suspected Barrett’s esophagus or significant mucosal abnormalities.

The high rate of chronic PPI use > 6 months in our cohort (73.4%) suggests that many patients require therapy beyond currently recommended intervals. Existing guidelines recommend empiric acid suppression in the immediate post-POEM period, with continuation until at least 3–6 months prior to reevaluation [6]. At our institution, long-term PPI therapy > 6 months is generally reserved for patients with objective evidence of pathologic acid exposure on Bravo testing. Although some patients remain on PPIs for symptom-directed management, our findings in this study support prior evidence demonstrating poor correlation between reflux symptoms and objective acid exposure after POEM. Notably, reflux esophagitis and abnormal acid exposure may lessen over time, suggesting progressive healing and remodeling of the LES [6]. Additionally, PPI therapy may become more effective with longer follow-up; Shiwaku et al. found improved PPI efficacy for esophagitis at 5 years compared with 1 year, and that symptomatic GERD incidence decreased over time among PPI users [11]. Identifying patients at higher risk for persistent symptoms may help guide surveillance intensity. In our cohort, older age and preoperative weight loss ≥ 5 kg predicted reflux symptom control on multivariable analysis, aligning with prior findings that younger age is associated with symptomatic reflux following POEM [23].

Twenty-three patients (29%) required additional procedures for achalasia during follow-up, reflecting the chronic nature of this disease. While POEM demonstrates durable efficacy for dysphagia relief, with long-term clinical success rates exceeding 90% at 5 years [23, 24], a subset of patients requires repeat intervention. Notably, patients in our study who underwent additional procedures were significantly less likely to have GERD-HRQL scores ≥ 10 (64.0% vs 90.5%, *p* < 0.001). Concurrent dysphagia (Eckardt score > 3) was present in 52.4% of patients with GERD-HRQL ≥ 10, compared to only 18.9% of those with adequate reflux control (*p* = 0.002). This association suggests that post-POEM symptom burden is often multifactorial, and patients with persistent reflux symptoms warrant comprehensive evaluation for both reflux and dysphagia rather than empiric escalation of acid suppression alone.

This study has several limitations. First, the 25.4% survey response rate introduces potential selection bias. While somewhat lower than mean rates reported in surgical survey literature [25], this likely reflects our prolonged follow-up period of 68 months, which itself represents a key strength enabling assessment of long-term outcomes. Second, the retrospective identification of our cohort with prospective survey follow-up may have introduced recall bias in symptom reporting. Third, the GERD-HRQL questionnaire was not administered preoperatively in our cohort, so we were unable to compare pre- and post-POEM scores. No standardized threshold exists for clinically significant reflux symptoms based on the GERD-HRQL questionnaire; however, our threshold of 10 is supported by anti-reflux surgery literature demonstrating that successfully treated patients typically achieve scores below this value, and the median score of 10 in our cohort provided a natural inflection point [13]. Fourth, Bravo pH monitoring was not performed uniformly in all patients, limiting the ability to correlate reflux symptoms with objective findings across the entire cohort. Finally, our single-center study may limit generalizability.

Despite these limitations, this study provides important long-term data demonstrating that reflux symptoms are prevalent years after POEM, with many patients requiring chronic PPI therapy. Reflux symptoms were incongruous with objective findings, highlighting the complexity of post-POEM surveillance and supporting the need for guideline recommendations for routine endoscopic and Bravo pH monitoring in all patients. Ongoing investigation should identify modifiable predictors of reflux, optimize surveillance protocols, and develop targeted strategies to mitigate post-POEM reflux burden.

## Supplementary Information

Below is the link to the electronic supplementary material.

Supplementary material 1 (DOCX 19 kb)
