# Supplementary material for: Reflux symptom burden and objective findings at long-term follow-up after peroral endoscopic myotomy
Source: Surg Endosc. 2026 Aug 3;40(10):9041–9. doi: 10.1007/s00464-026-13205-6 (PMC13633321; doi:10.1007/s00464-026-13205-6)
Supplement: Supplementary file 1 [file 464_2026_13205_MOESM1_ESM.docx]

**Eckardt Score Questionnaire**

1. Since your procedure, how often have you had difficulty swallowing?

Never ____ **(0)** Occasionally ____ **(1)** Daily ____ **(2)** With every meal ____ **(3)**

2. Since your procedure, how often have you felt like some of the food you swallowed was regurgitated, or “came back up” without being digested?

Never ____ **(0)** Occasionally ____ **(1)** Daily ____ **(2)** With every meal ____ **(3)**

3. Since your procedure, how often have you experienced chest pain?

Never ____ **(0)** Occasionally ____ **(1)** Daily ____ **(2)** With every meal ____ **(3)**

4. Since your procedure, have you lost any unintentional weight?

None ____ **(0)** Less than 11 lbs **(1)** Between 11 and 22 lbs **(2)** More than 22 lbs **(3)**

**TOTAL SCORE: ________**  [For internal use only]

The next set of questions are all designed to be answered according to a five-point scale as follows:

Please answer “0” if you have no symptoms; “1” if your symptoms are noticeable, but not bothersome; “2” if your symptoms are bothersome, but not every day; “3” if your symptoms are bothersome every day; “4” if your symptoms affect your daily activities; and “5” if your symptoms are incapacitating and cause you to be unable to perform daily activities.

| **GERD Health-related Quality of Life Questionnaire** | | | | | | | |
| --- | --- | --- | --- | --- | --- | --- | --- |
| 1. How bad is your heartburn? 2. Do you have heartburn while lying down? 3. Do you have heartburn while standing up? 4. Do you have heartburn after meals? 5. Does heartburn change your diet? 6. Does heartburn wake you up from sleep? 7. Do you have difficulty swallowing? 8. Do you have pain with swallowing? 9. Do you have abdominal bloating or distention? 10. If you take reflux medications, does this affect your daily life?   **TOTAL SCORE: ________**  [For internal use only] | 0  0  0  0  0  0  0  0  0  0 | 1  1  1  1  1  1  1  1  1  1 | 2  2  2  2  2  2  2  2  2  2 | 3  3  3  3  3  3  3  3  3  3 | 4  4  4  4  4  4  4  4  4  4 | 5  5  5  5  5  5  5  5  5  5 |  |

**If patient has any of the symptoms on the Eckardt Score Questionnaire or the GERD Health-related Quality of Life Questionnaire:**

How long after your POEM did you first have any of the symptoms above?

The final set of questions pertain to treatments you may or may not have received since your POEM:

1. Have you had another upper scope since your POEM?
   a. If “yes,” do you know the findings of the scope?
2. Have you had a Bravo study since your POEM? This is a study during which a small capsule is placed in your esophagus to measure acid reflux.
3. Have you had any other procedures due to difficulty swallowing or reflux symptoms since your POEM?
   a. If “yes,” was this:
      i. Botox injections into lower esophagus
      ii. Esophageal dilation
      iii. Another POEM
      iv. Heller myotomy
      v. Esophagectomy
4. Have you been taking a PPI medication since your POEM?
